# Nonlinear hysteretic behavior and anchorage performance of *Betula platyphylla* roots under cyclic loading

**DOI:** 10.3389/fpls.2025.1688551

**Published:** 2025-11-05

**Authors:** Yijun Xue, Donghui Zhao, Zeyu Zhang, Shihan Yang, Shumin Lyu, Jun Li, Xiaodong Ji

**Affiliations:** ^1^ Department of Civil Engineering, Beijing Forestry University, Beijing, China; ^2^ Key Laboratory of State Forestry Administration on Soil and Water Conservation, Beijing Forestry University, Beijing, China; ^3^ China Academy of Building Research, Beijing, China; ^4^ China Academy of Building Research (CABR) Testing Center Co., Ltd., Beijing, China; ^5^ State Key Laboratory of Hydraulics and Mountain River Engineering, College of Water Resource and Hydropower, Sichuan University, Chengdu, China

**Keywords:** cyclic loading, nonlinear hysteretic behavior, anchorage performance, Bouc-Wen model, energy dissipation

## Abstract

**Introduction:**

Cyclic loads caused by natural factors such as strong winds are common in plant growth environments. Prolonged exposure to such loads can compromise the anchorage performance of plants. This study examines how cyclic loading influences the root anchorage of Betula platyphylla, a prominent tree species in northern China.

**Methods:**

A series of pull-out tests were performed on soil-embedded roots, including monotonic pull-out tests and 100 cycles of loading and unloading.

**Results:**

The research results show that under different cyclic load amplitudes, the peak bearing capacity is negatively correlated with the load amplitude. Energy dissipation in the root system increases with higher load amplitudes but decreases as the number of cycles increases. From the initial cycle to the 25th cycle, energy dissipation decreased substantially, with no further significant reduction observed between the 25th and 100th cycles. To more effectively capture the nonlinear hysteretic behavior of roots, an enhanced Bouc-Wen model was developed and successfully fitted to the force-displacement curves. The model accurately replicated the hysteresis loops and characterized the damage progression in root anchorage under cyclic loading.

**Discussion:**

These findings offer valuable insights into the mechanical stability of plant roots under repeated environmental stresses and provide a robust framework for modeling root anchorage performance in natural settings.

## 1 Introduction

The mountainous regions of northern China often suffer from severe soil erosion problems. In addressing this challenge, vegetation-based slope protection has emerged as a sustainable ecological measure (Kumar et al., 2024; Yazdani et al., 2024), complementing traditional engineering approaches to form an integrated strategy for soil erosion control. The roots of vegetation are crucial in this process, serving as a key link between the plant’s aboveground biomass and the soil matrix. Through mechanisms such as anchoring and reinforcement, roots generate friction and mechanical interlocking with soil particles, thereby enhancing soil resistance to erosion (Ennos, 2000; Wu, 2007; Mckenzie et al., 2013; Kolb et al., 2017), and improving slope stability (Reubens et al., 2007; Ghestem et al., 2011; Leung et al., 2015), ultimately mitigating soil loss (El Kateb et al., 2013; Zhao et al., 2020). Recent studies have identified wind as a primary source of mechanical stress on vegetation (Biddington, 1986; Grace, 1988; De Langre, 2008; Gardiner et al., 2016). Wind forces are progressively transmitted from the main stem to the root system, subjecting roots to cyclic loading (Gardiner et al., 2016). Additional sources of loading include traffic-induced vibrations (Yang et al., 2019). Such cyclic stresses can compromise the integrity of the root-soil interface (Sun et al., 2023), diminishing the plant’s anchorage capacity and undermining its effectiveness in soil stabilization and slope protection (Wang et al., 2023; Wang et al., 2024). Prolonged exposure to such loads can compromise the root anchorage performance of plants, typically assessed through uniaxial pullout resistance. However, in this study, we focus on the behavior of roots under cyclic loading conditions, which differs from the traditional pullout resistance by considering the effects of repetitive stress cycles on root anchorage performance. When cyclic loading exceeds the root system’s anchorage capacity, particularly in the case of trees, uprooting and toppling may occur (Gardiner et al., 2016). This process displaces significant volumes of soil, leading to accelerated erosion. Consequently, understanding the anchorage performance of roots under cyclic loading is critical for enhancing soil erosion resistance and bolstering slope stability in vulnerable regions.

The anchorage capacity of a root system can be defined as the extent to which roots resist and adapt to mechanical forces transmitted through the plant structure (Niklas, 1999; Stubbs et al., 2019). Traditionally, this capacity has been evaluated through monotonic pullout tests, which apply steadily increasing tensile forces until failure, capturing peak resistance under extreme loading scenarios such as landslides or mudflows (Ji et al., 2018; Zhu et al., 2022). However, such events are rare. In natural settings, roots are more commonly exposed to repeated cyclic loads, including wind-induced swaying and runoff-driven erosion. Representing these as cyclic loading offers a more realistic approach for assessing *in situ* anchorage performance.

Recent studies have shown that cyclic loading can significantly degrade root tensile strength and anchorage performance, leading to pronounced hysteresis and energy dissipation (Mattheck et al., 1997; Karimzadeh et al., 2021; Hu et al., 2024). For instance, Wu, Leung, and Boldrin (Wu et al., 2024) reported that pre-cyclic loading at 5% and 50% strain levels reduced tensile strength by 18.17% and 27.10%, respectively. Makarova et al. (1998) observed high incremental plastic strain during early load cycles in fine roots of beech and larch. O’sullivan and Ritchie (1993) found that differences between static and cyclic anchorage strength in spruce trees were largely attributed to energy dissipation. Marchi’s field experiments further corroborated that, under cyclic tensile loading, the root system plays a crucial role in energy absorption and dissipation (Marchi et al., 2024). The interaction between roots and soil can protect the roots by transferring part of the energy out of the roots through soil deformation, thereby effectively enhancing the resilience of the composite system (Li P. et al., 2022; Hao et al., 2023). Building on the aforementioned findings, analyzing the elastic-plastic strain and energy dissipation mechanisms of root systems under cyclic loading is essential for comprehensively understanding the mechanical function of plant roots.

While most existing root mechanical models are based on single pullout tests (, 2013; Root methods: a handbook, 2013), recent efforts have aimed to better capture the progressive failure and load redistribution within root systems. For instance, Michlmayr et al. (2012) and Schwarz et al. (2010) introduced fiber bundle models (FBM) that simulate root anchorage by treating roots as bundles of elements subject to random sliding or fracture under increasing load. Pollen and Simon (2005) applied such models within bank stability frameworks and found that FBMs outperformed traditional approaches in predicting root reinforcement effects. Building on this, Schwarz et al. (2012) developed the root bundle model (RBM) to characterize the force–displacement behavior of root pullout, later refining it into a probabilistic variant (RBMw) by incorporating the Weibull distribution to account for strength variability among individual roots (Schwarz et al., 2013).

Despite these advances, the model’s applicability is limited and it is not suitable for cyclic loading. To achieve a continuous description of the hysteretic curve of roots under cyclic loading, this study improved the Bouc-Wen model (Ismail et al., 2009) to describe the hysteretic behavior of roots in an analytical form, which has gained increasing recognition for its ability to capture a wide range of hysteretic behaviors in analytical form. The model has been successfully applied in simulating cyclic responses in reinforced concrete (Yu et al., 2016) and soils (Peng et al., 2018; Pain et al., 2020), owing to its ability to replicate a broad range of hysteretic loop shapes (Ikhouane et al., 2007). Given the observed similarities between the hysteretic behavior of roots and that of other materials under cyclic loading (Barros et al., 2000). The Bouc–Wen model offers a promising basis for describing root anchorage under repeated environmental stresses.

In summary, this research centers on the root system of Betula platyphylla, a representative plant species in northern China, to investigate the hysteresis effect of roots under cyclic loading conditions simulating natural environmental influences in a controlled laboratory environment. By applying periodic loading-unloading with varying amplitudes to root systems embedded in soil, the study aims to investigate the effects of loading amplitude on root damage and energy dissipation. Additionally, an improved Bouc-Wen model is employed to characterize the root system’s hysteresis behavior, thereby providing a theoretical basis for predicting plant anchoring performance under dynamic environmental conditions. These findings offer valuable insights into the mechanical resilience of plant roots and their adaptive responses to cyclic, with implications for soil reinforcement in dynamic environments.

## 2 Materials and methods

### 2.1 Study area

**Figure 1:**
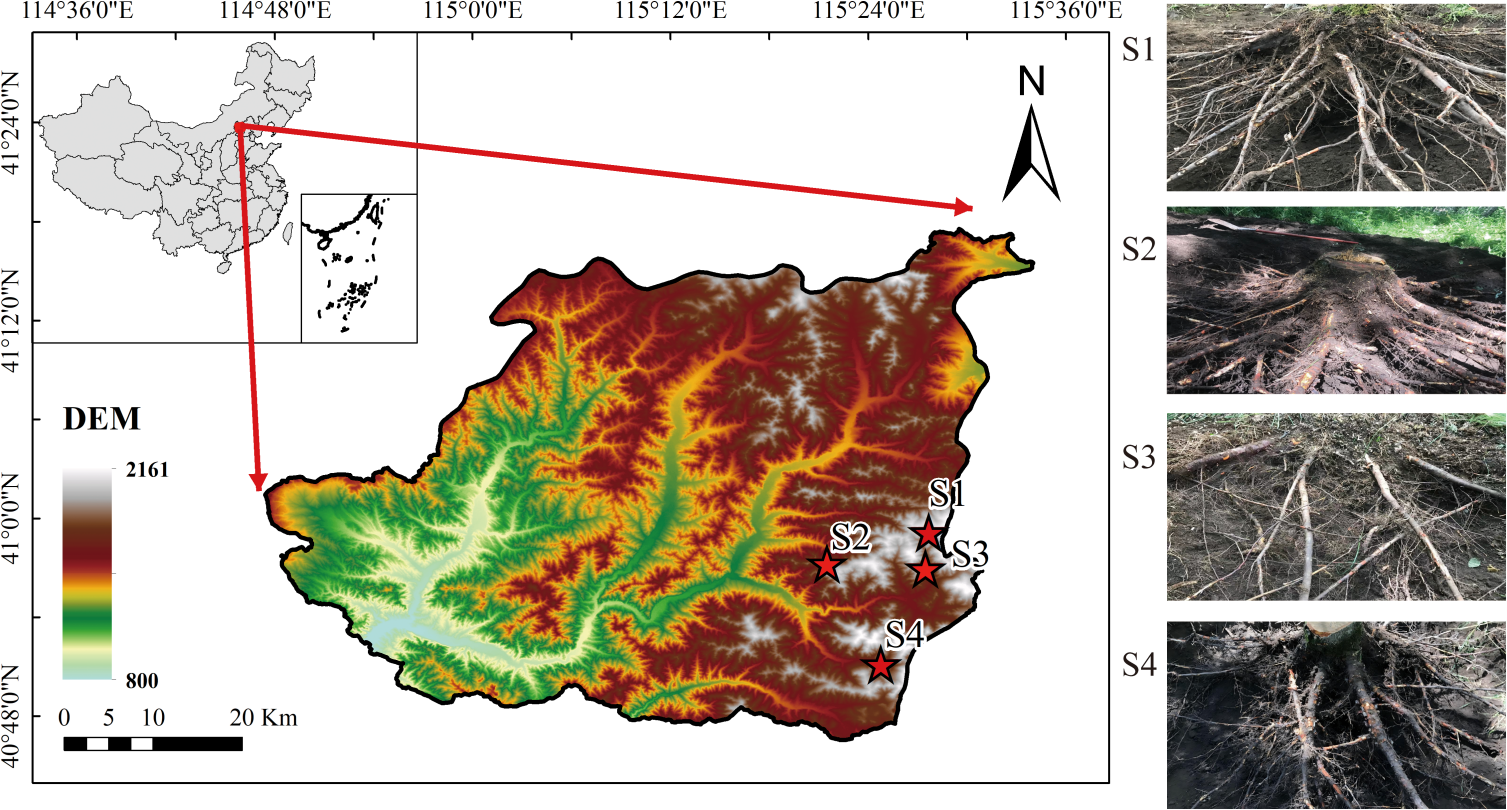
Study area location and sampled root system.

The study area is situated in the Taizicheng River basin within Chongli District, Zhangjiakou City, Hebei Province, covering a total area of 232.94 km². As illustrated in **Figure 1**, four sample points were selected and labeled as S1, S2, S3, and S4. The elevation of the area ranges from 1200 m to 2000 m, characterized by relatively rugged terrain. The region experiences an East Asian continental monsoon climate, where the average annual precipitation amounts to 482.4 mm and the annual average temperature is 3.81°C. Precipitation is highly unevenly distributed, with 75%–80% of the total annual rainfall occurring between June and September. The soil types in the study area are predominantly brown soils. Vegetation types fall within the warm temperate coniferous and deciduous broadleaf forest zone and the temperate grassland zone. The dominant vegetation is *Betula platyphylla*, with other tree species such as *Larix principis-rupprechtii*, *Picea wilsonii*, and *Armeniaca sibirica* also growing in the area. *Betula platyphylla* is widely distributed in northern China and is the dominant tree species in the study area. Previous studies have shown that *Betula platyphylla* roots exhibit superior tensile properties under similar conditions (Li X. et al., 2022). Furthermore, surveys within the study area revealed that *Betula platyphylla* forests had the highest soil fauna density and number of groups, as well as the highest Shannon-Wiener, richness, and evenness indices (Zhang et al., 2022). This suggests that *Betula platyphylla* forests possess high stability and diversity in their soil fauna, which could contribute to ecological restoration after slope planting. Overall, *Betula platyphylla* is the most suitable species.

### 2.2 Materials and sample preparation

In this investigation, Betula platyphylla, a naturally occurring secondary tree species prevalent located in the northwestern part of Hebei Province, was selected as the experimental subject. From each of the four designated sample plots, a single well-developed, 20-year-old Betula platyphylla tree was identified and designated as the standard tree for analysis. To ensure minimal disruption to the root system, the trees were felled horizontally, and the surrounding soil was meticulously excavated using the full excavation method until the root architecture was fully exposed. Roots with diameters smaller than 10 millimeter and devoid of visible damage were carefully selected, thoroughly washed. The USDA’s plant sampling guidelines (Johnson and Morgan, 2010) and references related to root sampling were (Delory et al., 2018; Freschet et al., 2021) were followed. In this experiment, after cleaning the roots, each birch root was placed in a sealed bag, de-aired, and sealed to prevent drying. The bag was then placed in a portable incubator. The bottom of the incubator was filled with ice packs, separated by foam boards. The sealed roots were placed on the foam boards, and the incubator was closed. The temperature inside the incubator was maintained at a constant 4°C to ensure temperature stability during transportation. After sampling was completed on the same day, the samples were quickly transferred to a laboratory refrigerator set at 4°C. Experiments were completed within three days of sampling to avoid prolonged storage and ensure sample stability.

Soil sampling was conducted within a 1-meter radius of each standard tree, with the top 20 cm of soil removed to access the 20–100 cm depth range. The collected soil samples were sieved on-site to eliminate large stones and gravel, ensuring homogeneity. The sieved samples were then sealed and transported to the laboratory for comprehensive physical property analysis. The physical characteristics of the soil from each sample plot are detailed in **Table 1**, providing a quantitative basis for further interpretation.

**Table 1:** Physical properties of soil.

Sn	Soil moisture content (%)	Average soil moisture content (%)	Soil density (g/cm³)	Soil dry density (g/cm³)	Average soil dry density (g/cm³)
S1	14.67	13.83	1.77	1.54	1.58
S2	13.85		1.73	1.58	
S3	14.13		1.79	1.57	
S4	12.92		1.82	1.61	

### 2.3 Experimental apparatus

**Figure 2:**
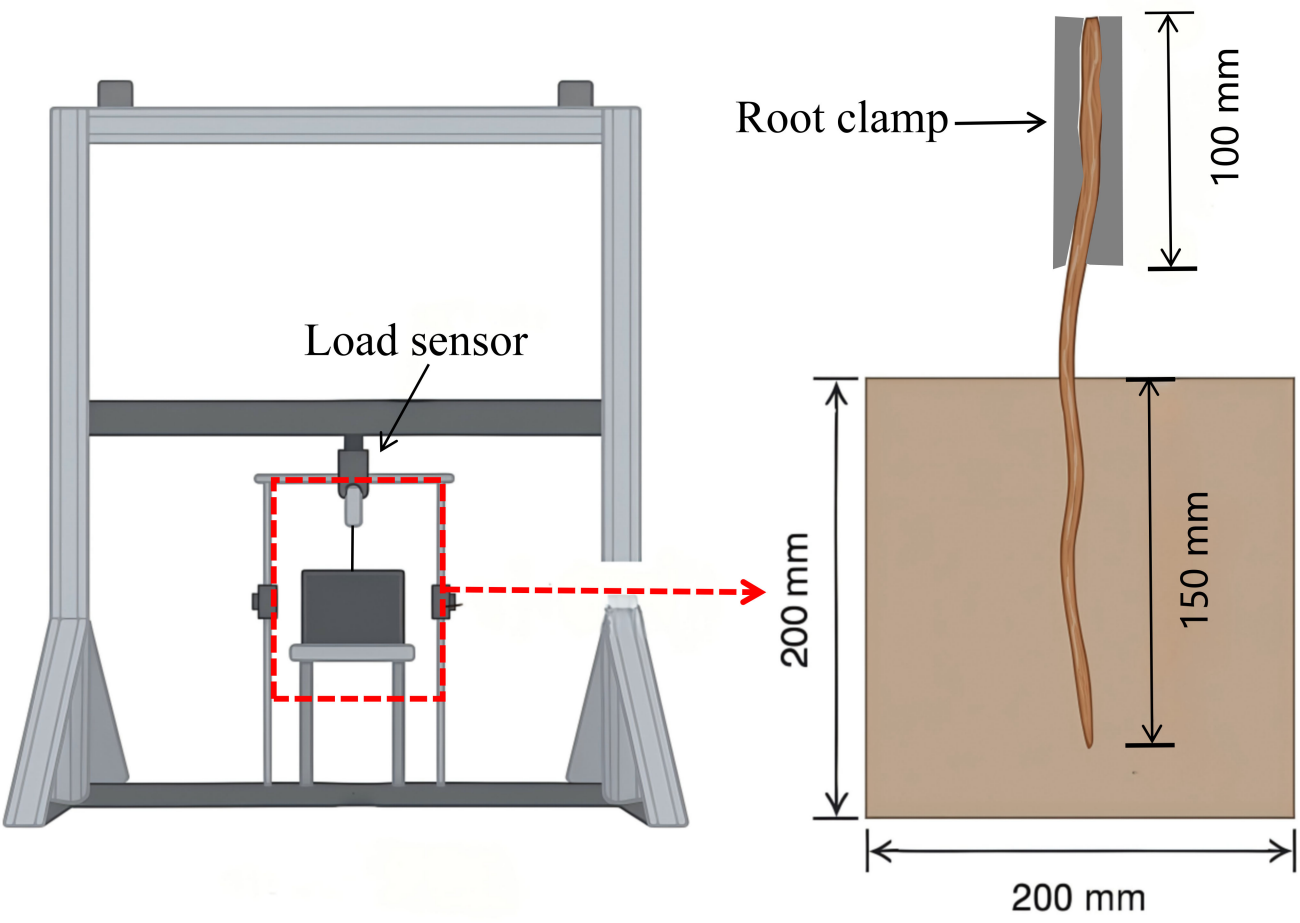
Root system pull-out testing machine and pull-out diagram.

In this study, a root pullout testing apparatus, independently developed by Beijing Forestry University, was employed to conduct the experiments (**Figure 2**). The apparatus is composed of three integrated systems: the loading system, the specimen system, and the data collection system. The loading system incorporates two synchronized servo motors and a vertical linear motion unit, both mounted on a crossbeam capable of vertical movement. This configuration ensures precise and controlled application of force during the pullout process. The specimen system comprises a steel box with sides measuring 200 mm and a root fixation device. The steel box features an open side to facilitate the filling of soil samples, while a rectangular aperture (42 mm × 10 mm) positioned directly above the box allows for the insertion and extraction of the system. The data collection system contains a load sensor (10 kN, 0–5 V output), a high-precision displacement sensor (LVDT) with an accuracy of 0.0001 mm, a data acquisition instrument, specialized data collection, a computer and analysis software. This system enables real-time monitoring and accurate recording of load and displacement data throughout the experiments.

### 2.4 Experimental procedure

In accordance with the experimental protocol, a soil sample was prepared with a controlled moisture content of 13.83%. The soil was introduced into the specimen box in five successive layers, following the principle of incremental filling to enhance compaction uniformity. Each layer was compacted using a 4 kg iron hammer and scarified to ensure effective interlayer bonding. During the filling of the third soil layer, a section of soil was initially added, after which the root system was carefully embedded, ensuring its central alignment within the soil matrix. The filling process was then resumed, and the soil was compacted to maintain structural integrity. The root system was buried to a depth of 15 cm and divided into three equal segments for analysis. The diameter of each segment of the root system was measured, and the average diameter was calculated. Root systems exhibiting a diameter variation exceeding 0.5 mm across the three segments were discarded and replaced to ensure uniformity. A 15 cm length of the root system was left exposed outside the specimen box. Of this, 10 cm was allocated for clamping and securing the roots, while the remaining 5 cm served as the free end length, ensuring consistent experimental conditions. The root sampling statistics are shown in **Table 2**.

**Table 2:** Root sampling statistics.

Sn	latitude and longitude	Average diameter (mm)	Number of test specimens
S1	115°27′41″E, 40°59′08″N	1.54-9.51	17
S2	115°21′30″E, 40°57′15″N	1.42-8.57	12
S3	115°27′29″E, 40°56′56″N	1.26-9.89	16
S4	115°24′47″E, 40°51′08″N	1.52-9.44	9
Total			54

Upon completion of the specimen preparation, monotonic tensile tests were first conducted. The specimen box was securely anchored to ensure vertical alignment between the root system and the clamping fixture. The root system was fixed in place, and a loading rate of 10 mm/min was applied to steadily extract the root. During this process, force and displacement data were continuously recorded, with the maximum pullout force defined as the peak bearing capacity. After the monotonic pullout test, roots that had not been pulled out during the monotonic pullout test were resampled and used to complete the cyclic loading test. In these tests, three upper-limit load levels, 25%, 50%, and 75% of the peak bearing capacity, were applied cyclically. Once the load reached the predetermined threshold, it was reduced to approximately zero. This loading–unloading sequence was repeated for 100 cycles, with the same pulling rate of 10 mm/min. If a root was pulled out before completing the set 100 cycles, the maximum pullout force was recorded as the peak bearing capacity. Conversely, if a root was not pulled out after completing 100 cyclic loading cycles, the root was pulled out monotonically at a rate of 10 mm/min to measure its peak bearing capacity, with the peak value recorded as the peak bearing capacity. This study aimed to analyze the changes in peak bearing capacity of the same root system before and after cyclic loading. In total, 54 individual Betula platyphylla roots were successfully tested, and their physical parameters are summarized in **Table 3**.

**Table 3:** Physical parameters of the roots.

Load amplitude	Average diameter (mm)	Number of root samples
25%	0.00-2.00	3
	2.00-4.00	4
	4.00-6.00	4
	6.00-8.00	4
	8.00-10.00	3
50%	0.00-2.00	3
	2.00-4.00	4
	4.00-6.00	4
	6.00-8.00	4
	8.00-10.00	3
75%	0.00-2.00	3
	2.00-4.00	4
	4.00-6.00	4
	6.00-8.00	4
	8.00-10.00	3

## 3 Results

### 3.1 Root damage after cyclic loading

After completing the monotonic pullout test, intact *Betula platyphylla* roots that were successfully pulled out were selected for the cyclic loading test. Considering the load amplitude factor, two types of damage patterns were observed in the roots during the pullout after cyclic loading. The first type of damage pattern (Pattern 1) occurred when the root system in the soil underwent 100 cycles of loading. After 100 cycles of cyclic loading, the root was completely extracted at a loading speed of 1 cm/min. The second type of damage pattern (Pattern 2) occurred when a portion of the root system failed to complete 100 cycles and was prematurely extracted. The details of these damage patterns are shown in **Table 4**.

**Table 4:** Root failure modes under different load amplitudes.

Load amplitude	Number of root samples	Destruction mode	
		Destruction mode 1	Destruction mode 2
25%	18	18	0
50%	18	18	0
75%	18	3	15

**Figure 3:**
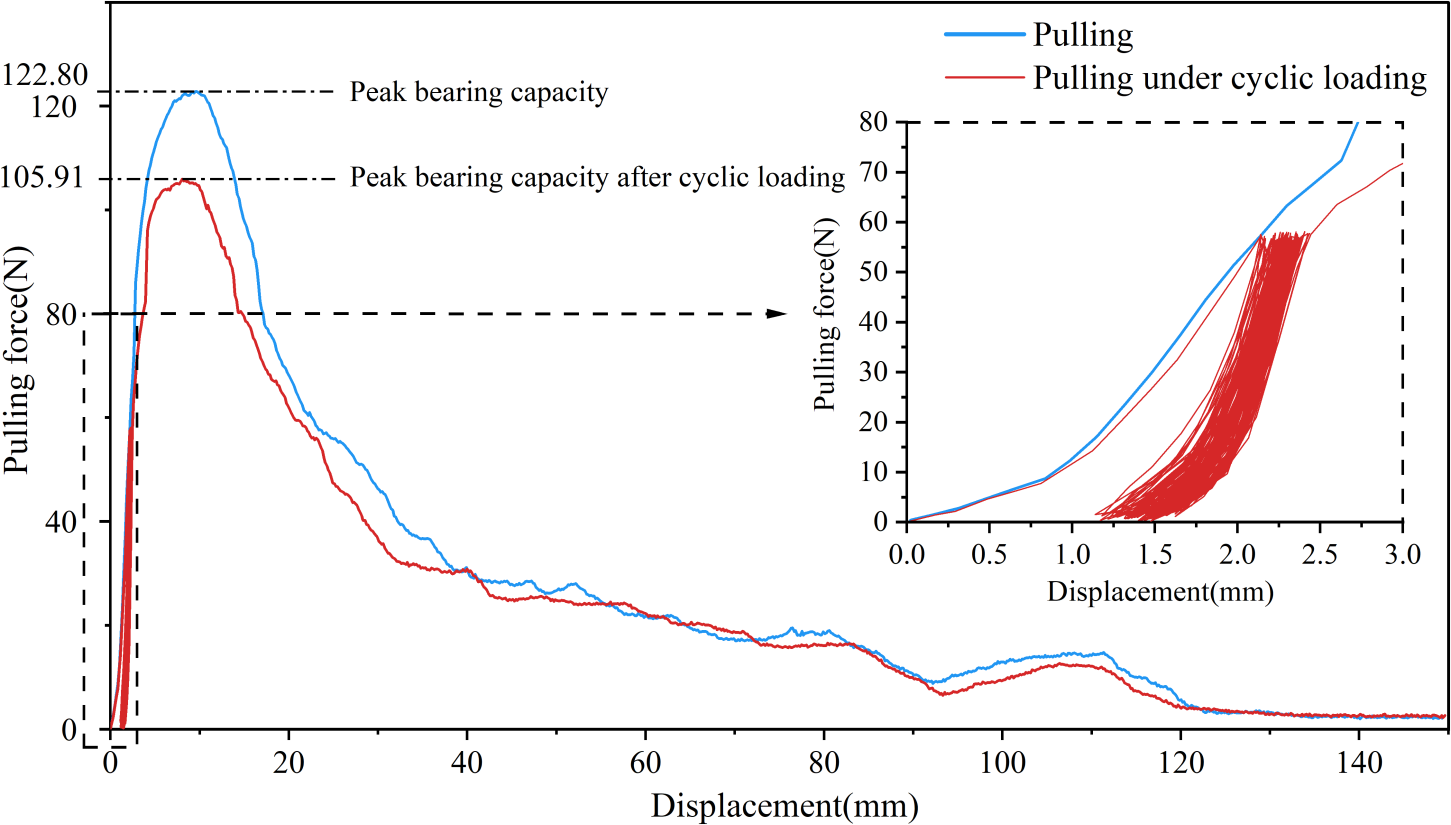
Force-displacement diagram for destruction mode 1.

**Figure 4:**
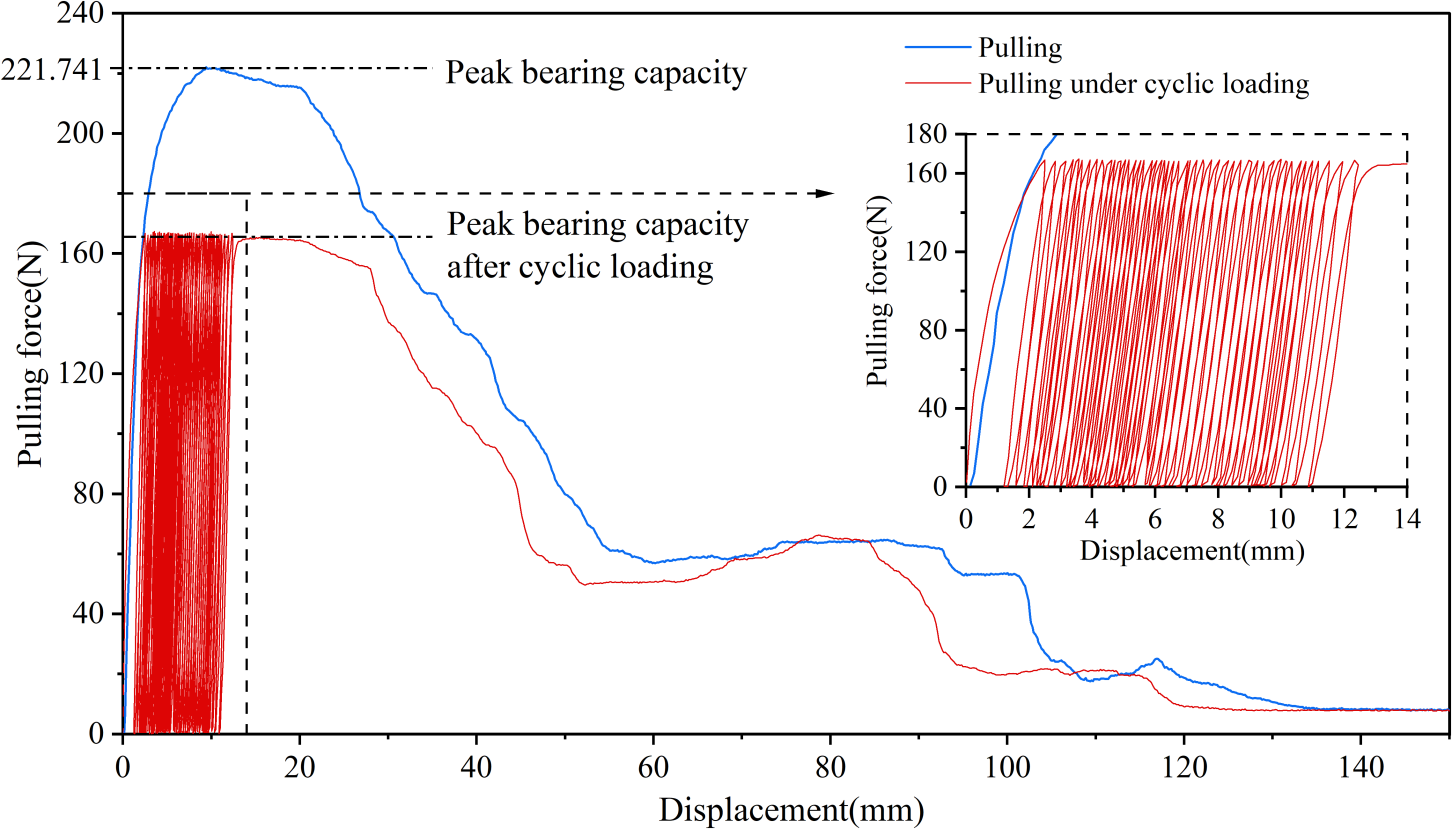
Force-displacement diagram for destruction mode 2.

Under the 25% and 50% cyclic load amplitudes, all 36 *Betula platyphylla* roots completed 100 cycles of cyclic loading and were pulled out in the form of damage pattern 1, and under 75% cyclic loading, three Betula platyphylla roots were pulled out in damage pattern 1. As shown in **Figure 3**, with the increase in the number of cyclic loading cycles, the total residual displacement continued to increase, while the increment of residual displacement per cycle gradually decreased. Additionally, the peak pullout force was lower than the peak pullout force after cyclic loading in a single pullout test. After undergoing 75% load amplitude cyclic loading, a total of 15 *Betula platyphylla* roots were pulled out, exhibiting damage pattern 2. As illustrated in **Figure 4**, the hysteresis curve of the root system under cyclic loading exhibited a more pronounced fusiform shape compared to the previous damage pattern. The hysteresis curve became sparse, then dense, and again sparse. The increment of residual displacement in the first half was similar to that of damage pattern 1, but in the second half, the increment of residual displacement increased with the number of cyclic loading cycles. When the number of cyclic loading cycles reached a certain value (but did not reach 100 cycles), during the final loading, before the pulling force reached the peak pullout force of the root, anchorage failure occurred and the root was pulled out. The pullout force, however, remained smaller than the peak pullout force in a single pullout test.

**Figure 5:**
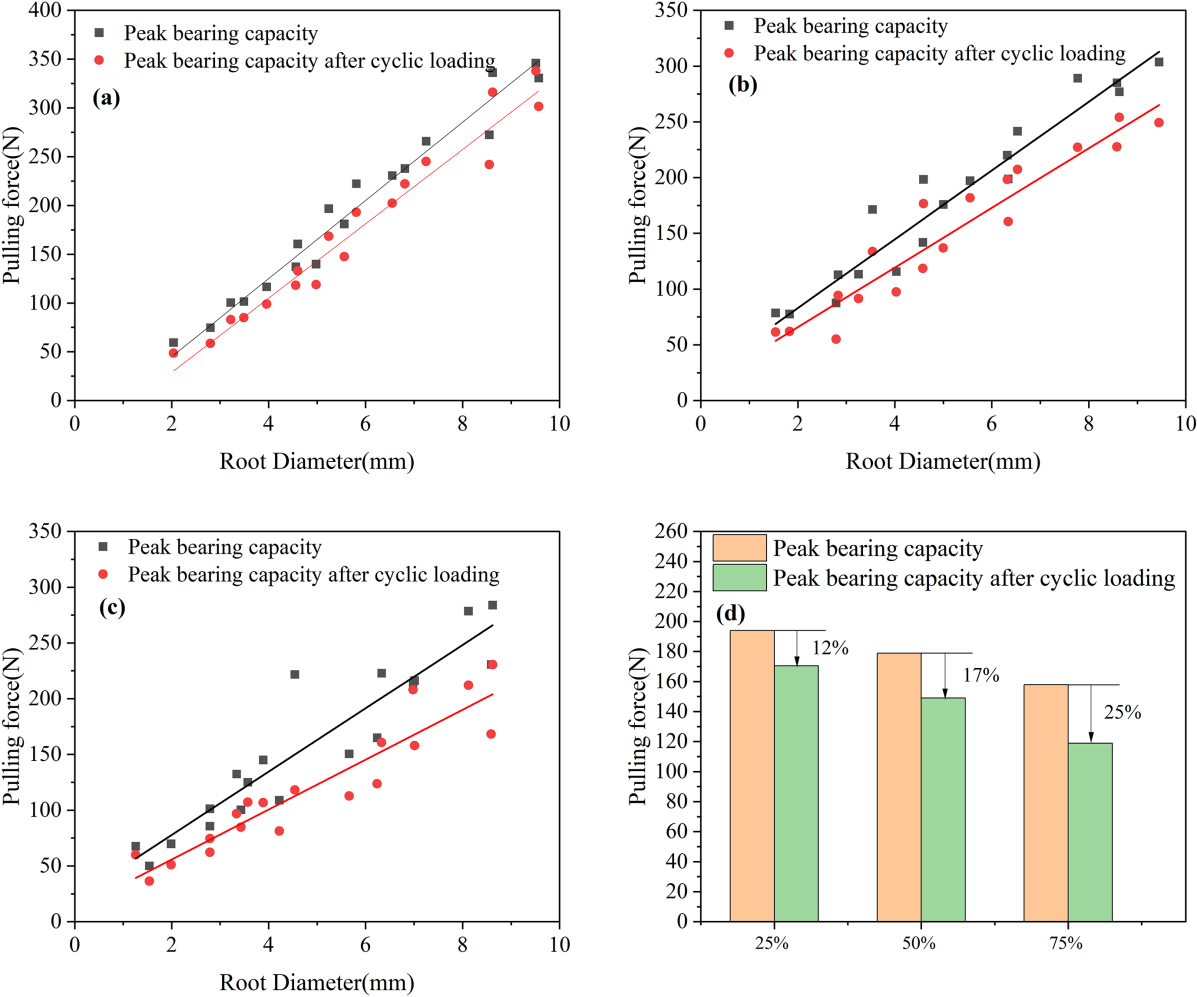
Peak bearing capacity before and after cyclic loading **(a)** 25% load amplitude **(b)** 50% load amplitude **(c)** 75% load amplitude **(d)** Percentage change in peak bearing capacity (%).

Comparing **Figures 5a-c**, it can be seen that the peak pullout force decreased after the application of 25%, 50%, and 75% cyclic load amplitudes. After 100 cycles of loading, the average peak pullout forces after the application of 25%, 50%, and 75% cyclic load amplitudes decreased by 23.40 N, 29.88 N, and 39.96 N, respectively. Compared to the peak pullout force before cyclic loading, the decreases were 12%, 17%, and 25%, as shown in **Figure 5d**. The data above indicate that with increasing load amplitude, the decrease in peak pullout force becomes more substantial, suggesting a greater impact on the anchorage performance of the root system.

Perform regression analysis on the curves in **Figures 5a-c**, as shown in **Table 5**. By comparing the regression equations of root peak bearing capacity and root diameter before and after cyclic loading, it can be seen that R^2^ gradually decreases, indicating that the correlation between peak bearing capacity and root diameter weakens after cyclic loading. By searching other literature, we found that the bearing capacity and root diameter also have the form of a power function (Zhang et al., 2020; Fan et al., 2025), but by comparison, it was found that the bearing capacity and root diameter are both positively correlated. Regarding the different fitting functions, this may be due to the influence of factors such as soil and root type. Therefore, based on the above results, we can draw a rigorous conclusion that the bearing capacity and root diameter are positively correlated, but the specific linear relationship may not apply to other experimental conditions.

**Table 5:** Regression equations for peak loads and diameters under different load amplitudes before and after cyclic loading.

Load amplitude	Cyclic loading	Regression equation	R^2^	P
25%	–	F = 38.95R-28.16	0.968	0.05
	Yes	F = 38.14R-45.19	0.957	0.05
50%	–	F= 30.66R + 23.87	0.930	0.05
	Yes	F = 26.58R + 14.29	0.906	0.05
75%	–	F = 28.50R + 19.93	0.867	0.05
	Yes	F = 22.52R + 10.82	0.861	0.05

### 3.2 Energy analysis of root system under cyclic loading

Cyclic load amplitude is an important factor influencing the evolution of the hysteresis curve. Therefore, the 25%, 50%, and 75% of the peak pullout force of the Betula platyphylla roots were used as the magnitudes of cyclic loading. Representative roots with diameters of approximately 1 cm, 3 cm, and 5 cm were selected to study the energy dissipation of the root system under cyclic loading. After 100 cycles of loading and unloading, the root loading-unloading force-displacement curves were obtained for different load amplitudes and root diameters.

**Figure 6:**
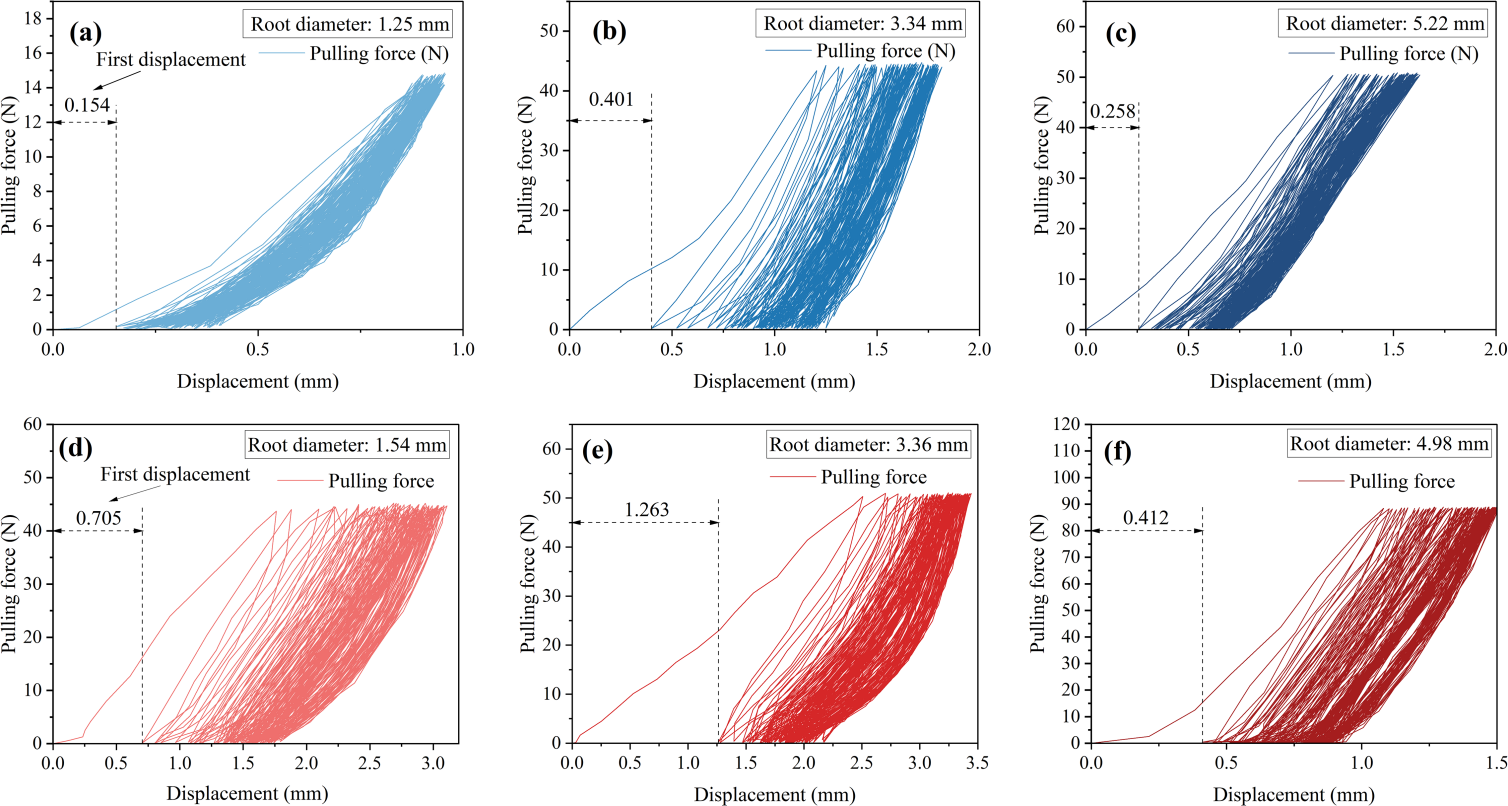
Root system cyclic load capacity-displacement diagram. **(a-c)** 25% cyclic load amplitude. **(d-f)** 50% cyclic load amplitude.

In the initial stage of 25% loading amplitude, as shown in **Figures 6a-c**, the first cycle of loading produced displacement responses of 0.301 mm, 0.401 mm, and 0.258 mm, respectively. Under the 50% cyclic load amplitude, the first displacement responses were 0.705 mm, 1.263 mm, and 0.495 mm, as shown in **Figures 6d-f**. From the above six figures, it can be observed that during the initial 1st to 3rd cycles, the hysteresis curve width was relatively wide, with significant energy dissipation. This clearly reflects the combined effect of energy storage and dissipation during the elastic-plastic deformation process of the root system. After multiple cycles of loading, the hysteresis curve transitioned from sparse to dense. The width of the hysteresis curve no longer significantly decreased. The additional displacement gradually decreased. Finally, the displacement increment approached zero, and the hysteresis curve tended to overlap.

**Figure 7:**
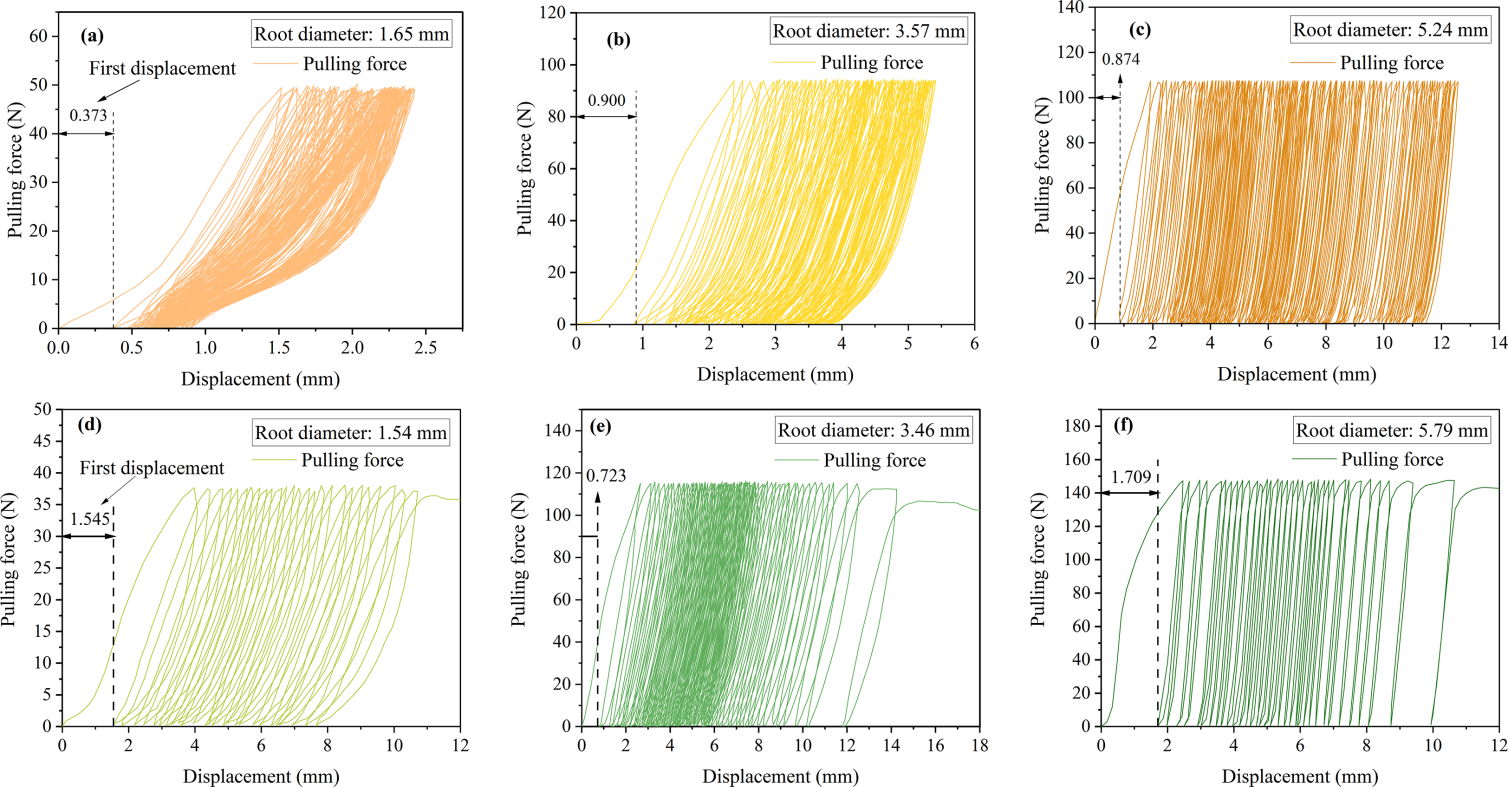
Root system cyclic load capacity-displacement diagram. **(a-c)** Roots not pulled out under 75% load amplitude. **(d-f)** Root pullout under 75% load amplitude.

The initial displacement responses under a 75% cyclic load amplitude were 0.373 mm, 0.900 mm, and 0.874 mm, respectively, as shown in **Figures 6a-c**. By combining the initial displacement responses under 25% and 50% cyclic loads, It is evident that as the cyclic load amplitude increases, the initial displacement response also increases. However, at a cyclic load amplitude of 75%, the root system responds to the initial load in the same way as at low load amplitudes. As the number of cycles increases, two distinct situations arise. The first type occurs when the hysteresis loop width does not change significantly. As the number of cycles increases, the displacement increment does not approach 0 but remains largely unchanged, ultimately completing 100 cycles of loading, as shown in **Figures 7a-c**. The second type involves a gradual widening of the hysteresis loop width, ultimately resulting in some roots being pulled out before reaching 100 cycles of load, as shown in **Figures 6d-f**. After the cyclic loading ended, the residual displacement was observed to be positively correlated with the amplitude of the cyclic load, with larger amplitudes resulting in greater residual displacement.

**Figure 8:**
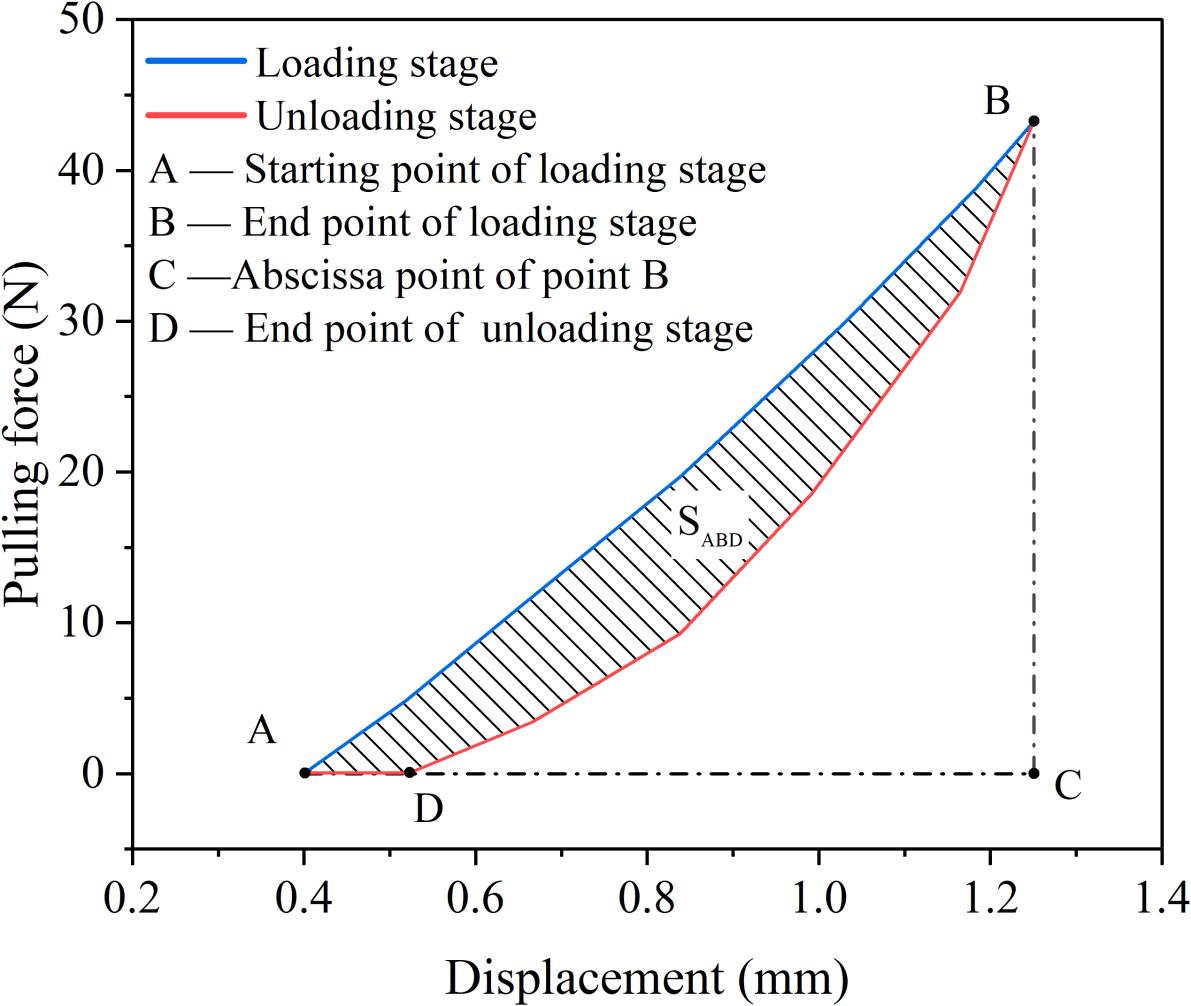
Schematic diagram of root energy consumption.

Under cyclic loading, the formation of the hysteresis curve can be further understood from the perspective of energy. The area enclosed by the hysteresis loop represents the energy dissipation within a single loading cycle. This energy primarily reflects the irreversible deformation occurring within the material, along with the associated damage and fatigue accumulation. As shown in **Figure 8**, during segment AB of the curve, the tensile loading phase, the work done by the external force on the root system corresponds to the area enclosed by curve ABC. Part of this work is stored as elastic potential energy within the root, while the remainder is dissipated through irreversible processes such as plastic deformation and root slippage, transforming into heat or other forms of energy dissipation. In segment BD of the curve, the unloading phase, the tensile force gradually decreases. During root unloading, the area bounded by curve DBC can be used to calculate the released elastic potential energy. Since energy dissipation is unidirectional and irreversible, the unloading curve of the root will always lie below the loading curve during return. The difference between the loading area and the unloading area, S_ABD_, represents the energy dissipated during this loading cycle, ultimately resulting in irreversible residual displacement, i.e., the length of segment AD. The difference in area of this closed region represents the energy dissipated during a single loading and unloading cycle, which is an important indicator of cumulative damage and fatigue in materials during cyclic loading.

**Figure 9:**
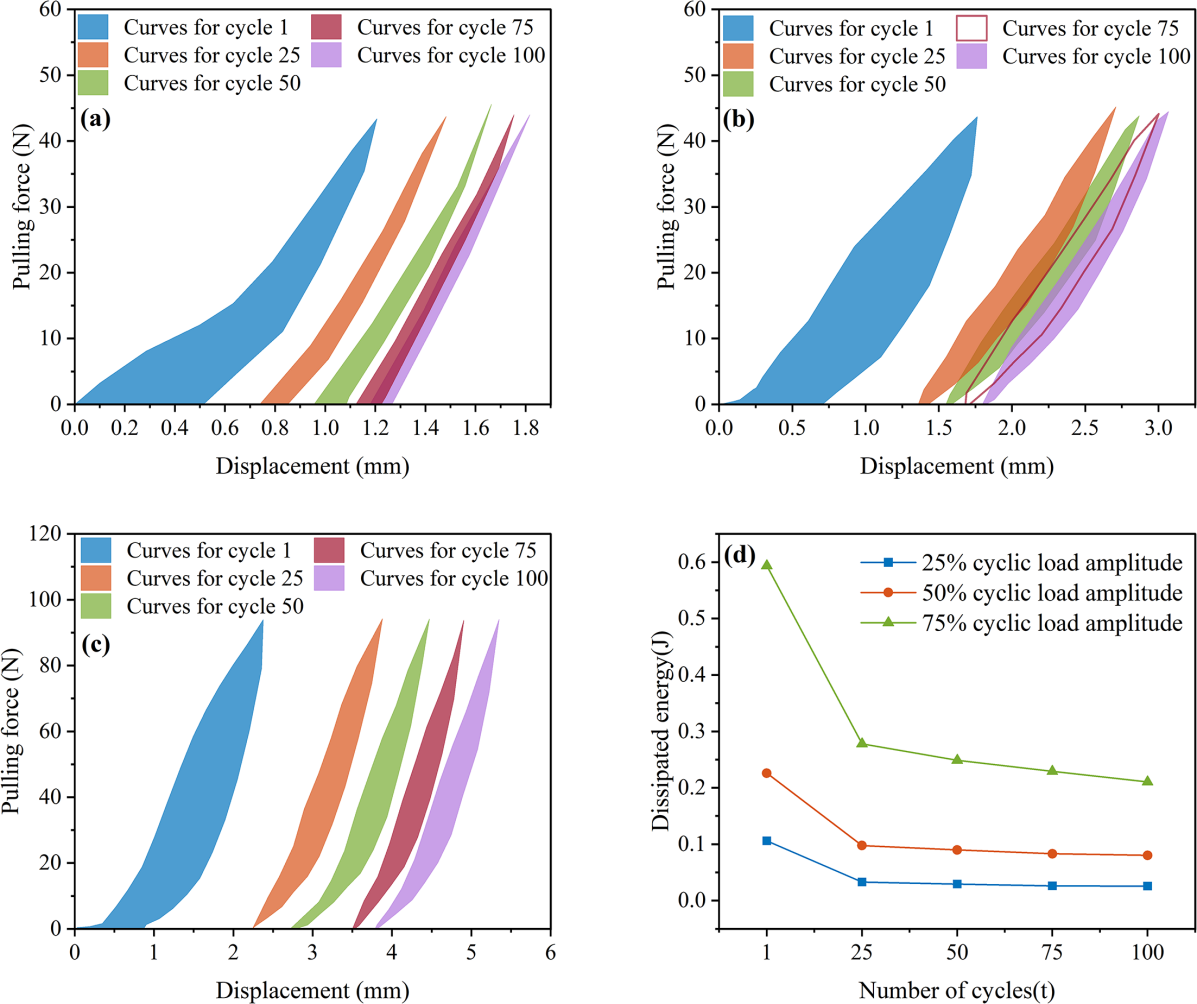
Energy consumption of root system cyclic loading **(a)** 25% load amplitude **(b)** 50% load amplitude **(c)** 75% load amplitude **(d)** Energy consumption trend.

**Figure 9a** shows the hysteresis curves for the first, 25th, 50th, 75th, and 100th load cycles under a 25% load amplitude. The area enclosed by the hysteresis curves is integrated to calculate the area of the first hysteresis curve, which is 10.5835 J, resulting in a dissipated energy of 0.106 J. Similarly, the energy dissipated during the 25th, 50th, 75th, and 100th load cycles was 0.0329 J, 0.0294 J, 0.02611 J, and 0.02575 J, respectively. At a load amplitude of 50% (**Figure 9b**), the dissipated energy for the 1st, 25th, 50th, 75th, and 100th cycles was 0.2259 J, 0.0977 J, 0.0899 J, 0.08303 J, and 0.0803 J, respectively. At a load amplitude of 75% (**Figure 9c**), the dissipated energy was 0.5935 J, 0.2777 J, 0.2488 J, 0.2291 J, and 0.2105 J, respectively. Summarizing the above data, it can be observed that as the load amplitude increases, the energy dissipated by the root system also increases. This is because a 75% load amplitude induces greater elastic-plastic deformation in the root and causes more significant damage to the root-soil interface, and a faster decay in root stiffness, all of which contribute to increased energy dissipation. At the same time, under the same load amplitude, the more cycles there are, the less energy is dissipated, as shown in **Figure 9d**. The energy dissipation curve exhibits a clear downward trend with the number of cycles. At load amplitudes of 25%, 50%, and 75%, the energy dissipated during the 25th load cycle decreased by 68.96%, 56.75%, and 53.21%, respectively, compared to the first cycle. This is because the root system undergoes elastic-plastic deformation for the first time, and the original root-soil interface is disrupted, leading to maximum energy dissipation.

### 3.3 Improved Bouc-Wen model under cyclic loading

In order to make the Bouc-Wen model more consistent with hysteresis curves under different conditions, some researchers have introduced additional parameters to refine the model based on characteristics observed in experimental curves. By improving the model to better reflect experimental data, these improvements have enhanced the model’s predictive capabilities, making it applicable to specific materials. In this study, after comparing the results of the Bouc-Wen model with those of root cyclic loads, shape parameters related to root deformation were incorporated to describe the hysteresis behavior of the root-soil interface, based on the hysteresis curve characteristics of the interface. Based on the Bouc-Wen model, the pull-out force under the root cyclic load can be expressed as Equation 1:


(1) F=kx+(1+q)(1−α)kz

In Equation 1: *F* is the tensile force; *k* is the initial linear elastic stiffness; α is the post-yield stiffness ratio; *q* is the shape parameter; *x* is the root displacement; *z* is the hysteresis displacement. The relationship between root displacement *x* and hysteresis displacement *z* is given by Equation 2:


(2) $$z=(1/\eta )[Ax-\nu (\beta |\dot{x}|\;|z{|}^{n-1}z+\gamma \dot{x}|z{|}^{n})]$$

In Equation 2, *A, β, γ* and *n* are shape parameters of the hysteresis curve.

*A* is the amplitude of the hysteresis loop, determining the extent of deformation during loading and unloading cycles.

*β* is a parameter controlling the sharpness of the transition between elastic and plastic deformation, influencing the shape of the force-displacement curve.

*γ* represents the rate of energy dissipation during the cyclic loading process, with higher values leading to greater energy loss.

*n* is the power exponent that characterizes the nonlinearity of the material’s response to loading, determining how the system’s stiffness changes with increasing strain.


(3) $$\eta =1-\delta_{\eta }\epsilon ,\;\nu =1-\delta_{v}\epsilon$$

In Equation 3: $\delta_{\eta }$ and $\delta_{\nu }$ are the stiffness and strength degradation rates, respectively;


(4) $$\epsilon =(1-\alpha )k\int_{0}^{T}xzdt$$

In Equation 4: *ϵ* is the cumulative hysteretic energy dissipation within the time history *T*.

The shape parameters in the above model lack quantitative calculation formulas. Therefore, this paper employs Matlab for programming and combines two optimization algorithms, namely the differential evolution (DE) algorithm and the particle swarm optimization (PSO) algorithm, to identify the parameters in the model and obtain the optimal parameter values for the improved Bouc-Wen model based on the best-fitting results. The differential evolution (DE) algorithm excels at handling complex, multi-peak nonlinear optimization problems, while the particle swarm optimization (PSO) algorithm is simple to implement and converges quickly. The optimization process is terminated when the fitness function value reaches the preset error limit, thereby completing the parameter identification for the improved model. Next, MATLAB programming is used to plot the actual loading and unloading force-displacement curves along with the model’s force-displacement curves.

Based on loading data obtained under three different cyclic load amplitudes of 25%, 50%, and 75%, a total of 54 specimens were used to identify the parameters of the improved Bouc-Wen model through the application of an optimization algorithm. Due to the large volume of data, this table presents four representative cases under three amplitude conditions. The parameter identification values for each case are shown in **Table 6**, while the remaining samples are used to evaluate the model’s accuracy. By statistically analyzing 54 root samples to identify the parameters of the improved Bouc-Wen shape parameter q, we concluded that the parameter identification range of *q* is 0-2. As the number of iterations increases, the range of *q* gradually decreases, ultimately yielding an accurate *q* value.

**Table 6:** Improved Bouc-Wen model parameter identification values.

Sample	α	k	q	A	β	γ	n	${\text{δ}}_{\text{η}}$	${\text{δ}}_{\text{ν}}$
1	0.0742	8.4698	1.8000	0.9965	0.8818	3.9308	0.0010	0.0033	1.8000
2	0.3990	17.4611	0.4714	0.7224	0.9056	0.0110	3.2843	0.0010	0.0675
3	0.1646	50.9369	0.7324	0.6900	-0.2273	1.0103	-0.0019	-0.0021	0.2000
4	0.0079	145.9889	0.00001	0.7700	0.1935	0.1084	9.8437	0.00000001	0.00000001

**Figure 10:**
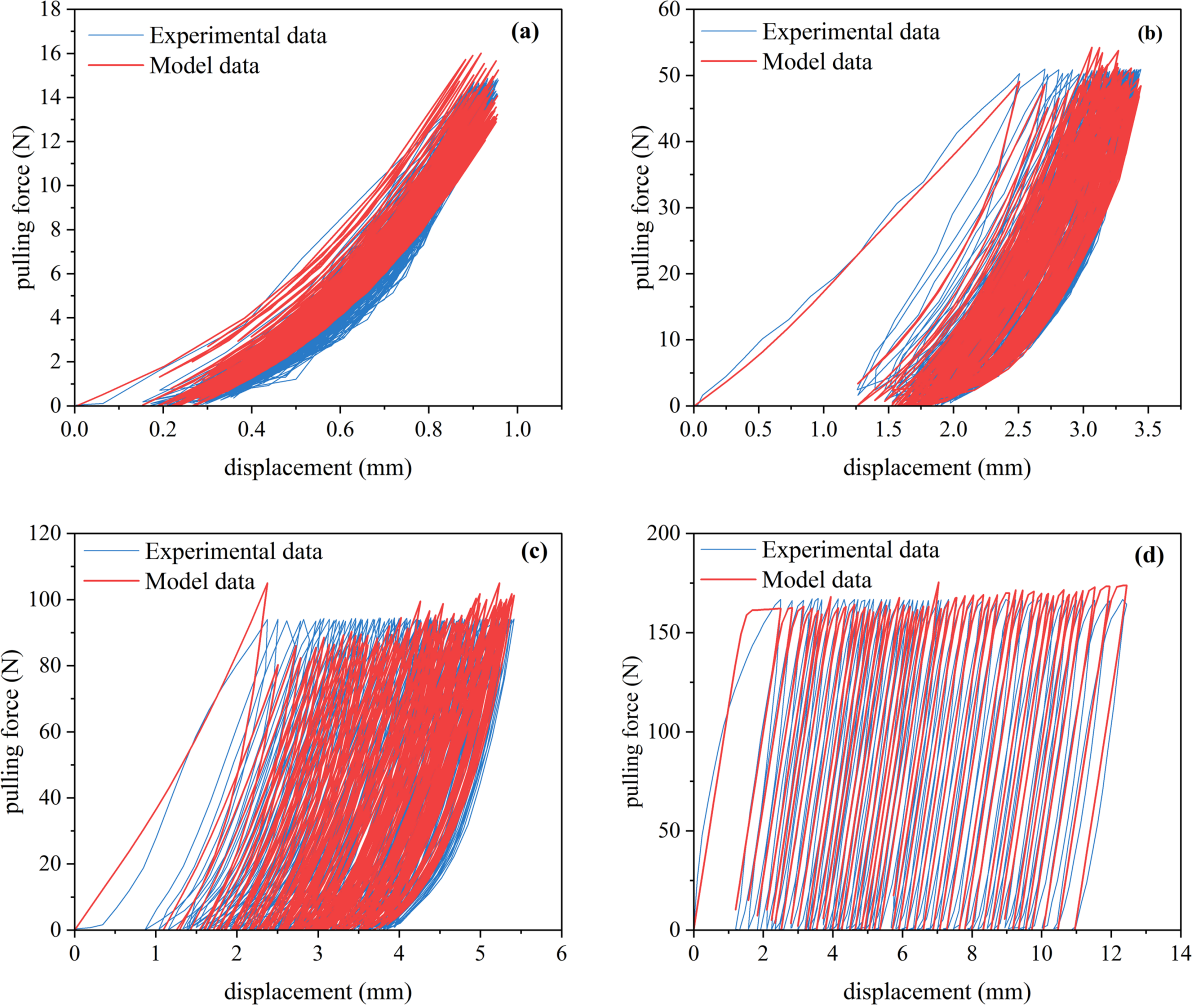
Model fitting results **(a)** 25% load amplitude **(b)** 50% load amplitude **(c)** Roots not pulled out under 75% load amplitude **(d)** Roots pulled out under 75% load amplitude.

The hysteresis curves determined by the improved BoucWen model and the experimental data values are shown in **Figure 10**. In **Figures 10a-c**, the root systems were not pulled out after undergoing 100 cycles of loading. However, in **Figure 10d**, the root system was pulled out before reaching 100 cycles under 75% cyclic loading. Nevertheless, the improved Bouc-Wen model was still able to accurately fit the hysteresis behavior of the root systems described above. The results indicate that the improved root-soil cyclic load model, based on the Bouc-Wen model, shows a high degree of agreement with the experimental data. It captures the main characteristics of the hysteresis curve and effectively describes the hysteresis behavior of roots under cyclic loads.

**Figure 11:**
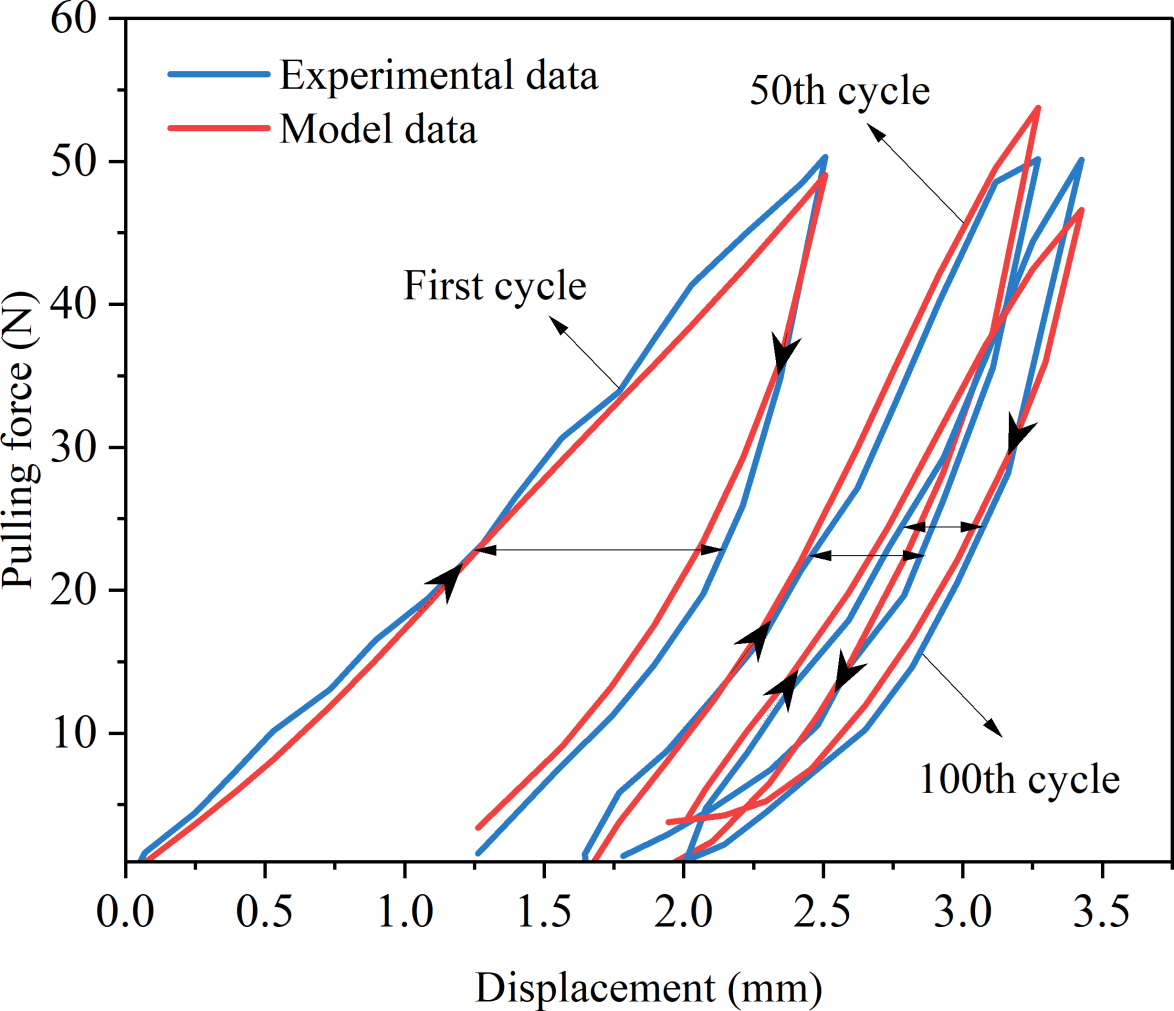
Schematic diagram of model fitting results.

In order to better demonstrate the accuracy of the fitting results, we drew a schematic diagram of the fitting results, as shown in **Figure 11**.

In order to further evaluate the fitting of the improved Bouc-Wen model, this study decided to use the goodness-of-fit index to evaluate the improved Bouc-Wen model. The coefficient of determination (R²) is a measure of goodness of fit, and its formula is given by:


(5) $$R^{2}=1-\frac{\sum_{i-1}^{n}{\left(F_{i}-\hat{F_{i}}\right)}^{2}}{\sum_{i-1}^{n}{\left(F_{i}-\bar{F_{i}}\right)}^{2}}$$

In Equation 5, Fi is the i-th empirical cumulative distribution function value, represents the corresponding cumulative distribution function estimate, and is expressed as the average value of Fi. The R^2^ evaluation index is used to assess the accuracy of the improved Bouc-Wen model. The index value ranges from 0 to 1, with values closer to 1 indicating smaller relative prediction errors and better model performance.

**Figure 12:**
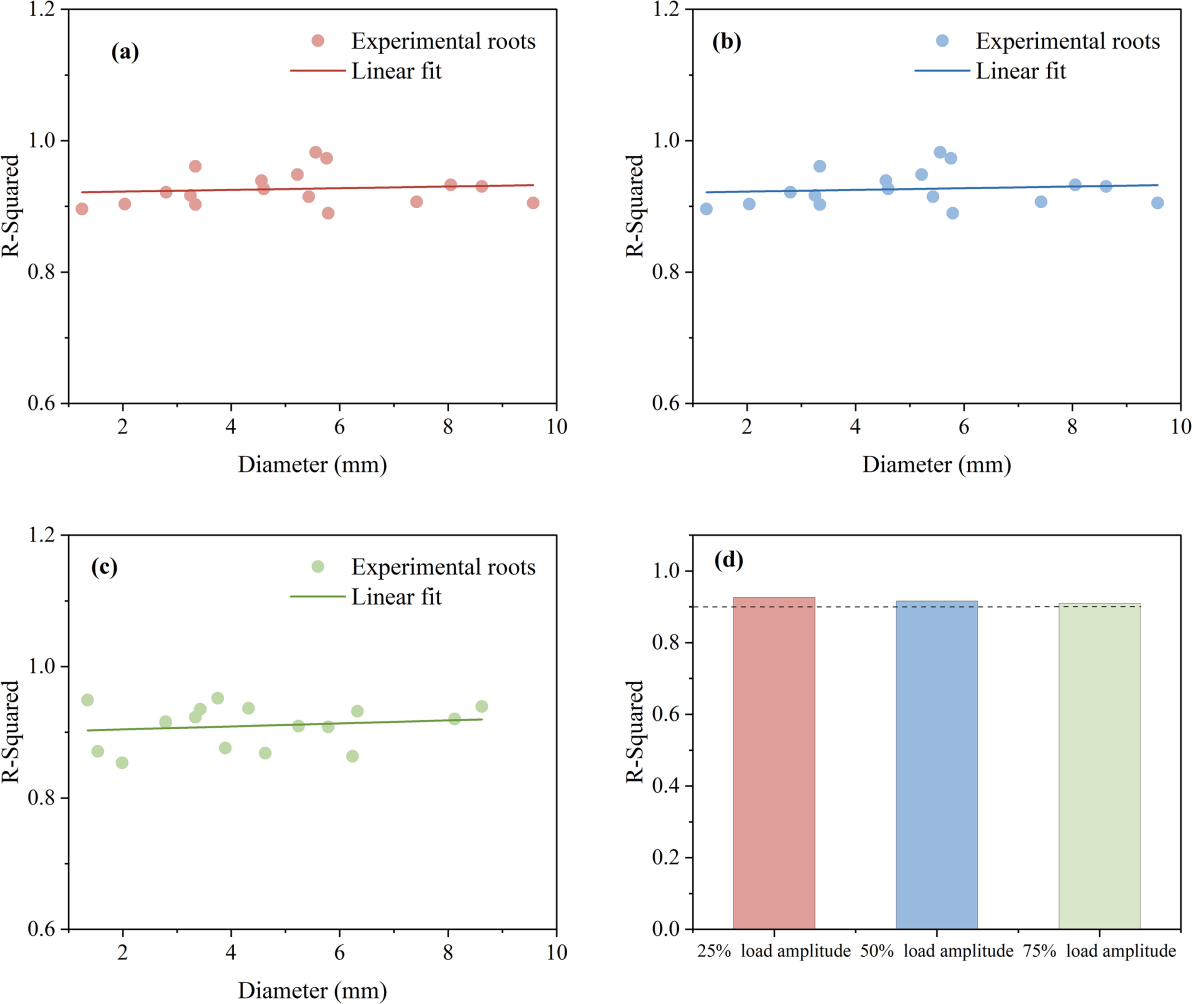
Model accuracy **(a)** 25% load amplitude **(b)** 50% load amplitude **(c)** 75% load amplitude **(d)** Average coefficient of determination.

To further assess the effectiveness of the improved Bouc-Wen model, the coefficient of determination (R²) was used to evaluate the accuracy of the model. As shown in **Figure 12**, the root sample diameter ranges from approximately 1 to 10 cm, and the model’s accuracy is evaluated under three different load amplitudes. In **Figure 12a**, the model fits well with a 25% load amplitude, with the average R² for the 18 data sets being 0.927. In **Figure 12b**, the average R² for the 18 data sets under a 50% load amplitude is 0.916. In **Figure 12c**, under a 75% load amplitude, the average R² for the 18 root system data sets is 0.910. Overall, the Bouc-Wen model demonstrated high accuracy for the force-displacement curves of root cyclic loading and unloading under three different load amplitudes, as shown in **Figure 12d**, with R² exceeding 0.9 and an average R² value of approximately 0.917.

## 4 Discussion

This study extends previous research on the mechanical behavior of root systems under cyclic loading, refining both experimental design and data analysis methodologies. Comprehensive experiments were conducted across varying load amplitudes (25%, 50%, 75%) and cycle numbers (100 cycles), yielding more detailed conclusions, particularly regarding root damage mechanisms and energy dissipation. This study also revealed the nonlinear mechanical response of root systems under dynamic load conditions. A key innovation of this work is the introduction of an enhanced Bouc-Wen model, which more accurately simulates and predicts the nonlinear hysteretic behavior of root systems under repeated loading, thereby laying a theoretical foundation for the further advancement of plant root mechanical models.

### 4.1 Root damage after cyclic loading

The findings reveal that *Betula platyphylla* roots exhibit distinct failure mechanisms under varying cyclic loading amplitudes. At amplitudes of 25% and 50%, all roots sustained 100 loading cycles and damage in pattern 1, characterized by the progressive accumulation of residual displacement with diminishing increments per cycle, alongside a lower peak pullout force compared with single-pullout tests. These observations suggest that under low- to moderate-amplitude disturbances, the root–soil interface is governed by gradual interfacial slip and localized soil compaction. Such processes induce particle rearrangement, mechanical interlocking between roots and soil, and an increase in frictional adhesion (Schwarz et al., 2010; Bengough et al., 2011), ultimately leading to system stabilization. This behavior is consistent with the structural rearrangement and strain-hardening response of granular media subjected to repeated loading (Desrues and Andò, 2015) and corroborates prior observations of the gradual attenuation of frictional resistance at the root–soil interface (Yang et al., 2022; Lv et al., 2025). In contrast, under 75% cyclic loading amplitude, roots predominantly exhibited damage pattern 2. This indicates that high-amplitude loading markedly accelerates interface degradation: soil aggregates contributing to interlocking were disrupted under elevated pullout forces, the root–soil interface became smoother, and frictional bonding progressively weakened, culminating in interface bond failure and complete root extraction. Comparable anchorage failures have been reported in trees (Kamimura et al., 2012). Together, these results elucidate the amplitude-dependent failure mechanisms governing root anchorage.

The peak bearing capacity following cyclic loading serves as a critical metric for evaluating root anchorage performance. In this study, *Betula platyphylla* roots subjected to cyclic loading with amplitudes of 25%, 50%, and 75% exhibited a marked decline in peak bearing capacity, accompanied by a reduction in anchoring performance. These findings align with the experimental results reported by O’Sullivan, Yang S et al (O’sullivan and Ritchie, 1993; Yang et al., 2023). Notably, cyclic loading induces root damage in a manner analogous to monotonic tensile loading. Research has demonstrated that cyclic loading triggers microstructural alterations in root systems. During the initial loading phase, cell wall stress reaches its maximum, followed by stochastic decay in cell wall strength (McLaughlin and Pitt, 1984; Tenhaken, 2015). As cyclic loading progresses, the cell wall structure undergoes further modifications (Shah, 2016), with rupture propagating along a plane perpendicular to the applied stress axis. Consequently, the peak bearing capacity of the root system diminishes under cyclic loading.

In this experiment, under cyclic load amplitudes of 25% and 50%, roots were completely extracted after 100 loading-unloading cycles, with peak bearing capacity decreasing by 12.07% and 16.73%, respectively. Under a 75% cyclic load, three roots were pulled out after completing 100 cycles, while fifteen roots failed before reaching 100 cycles. Fatigue theory in materials science offers valuable insights into this behavior. Basquin’s law (Kun et al., 2008) posits that the fatigue life of a material under cyclic loading follows a power-law relationship with stress amplitude; higher stress amplitudes correspond to shorter fatigue lifetimes. Numerous studies (Yang and Nayeb-Hashemi, 2006; Herrmann and Kun, 2007; Herrmann et al., 2009) have Basquin’s law using fibrous materials, suggesting its applicability to plant root systems. For a given number of cycles, higher cyclic load amplitudes result in more pronounced root damage and a greater reduction in peak bearing capacity. Thus, a negative correlation exists between cyclic load amplitude and peak bearing capacity, underscoring the detrimental effects of increased loading intensity on root mechanical performance.

### 4.2 Energy dissipation in root systems under cyclic loading

Like most plants, the root system of *Betula platyphylla* exhibits both elastic and plastic properties. From an energy perspective, cyclic loading induces root deformation (Potocka and Szymanowska-Pułka, 2018), and disrupts the root-soil interface. Consequently, energy dissipation during cyclic loading occurs primarily through the elastic-plastic deformation of the root system and friction at the root-soil interface.

From the perspective of elastic-plastic deformation, part of the external force applied to the roots induces elastic deformation, which is stored as elastic potential energy. A portion of this energy, however, causes plastic deformation in the roots, which is not recovered upon unloading. This results in a hysteresis loop, where the unloading curve lags behind the loading curve (Chun-juan et al., 2013). The root system’s tissue structure comprises the periderm, secondary phloem, and secondary xylem (Verdaguer and Molinas, 1999; Gričar et al., 2015), each with distinct mechanical strengths (Zhu et al., 2009). During the first few cyclic loads (cycles 1-5), repeated stretching causes the periderm, which has limited elastic deformation capacity, to fail first, leading to significant energy dissipation. As the number of cycles increases, the secondary phloem and secondary xylem mitigate root damage through fiber stretching (Li et al., 2018), causing energy dissipation to gradually decrease. Under sustained cyclic loading, plastic deformation shifts the equilibrium position of stress distribution within the root, resulting in uneven stress distribution. This reduces plastic deformation over time, slows damage accumulation, and leads to progressive root deterioration (Zhu et al., 2020). However, this energy dissipation may not always correlate directly with irreversible plastic deformation. As shown in studies such as Parafinuik et al (Parafiniuk et al., 2018), hysteresis can occur with plasticity under certain loading conditions, but also without plasticity, suggesting that the relationship between hysteresis and plastic deformation is more complex than previously assumed. This reversible slippage could potentially enhance the system’s toughness by diverting energy from the root system (Zhu et al., 2022). Overall, energy dissipation in the root system results from the combined effects of elastic and plastic deformations.

Simultaneously, from the perspective of root-soil interface friction, the initial pull causes the root system to adjust its shape and undergo plastic deformation, loosening the surrounding soil (Kolb et al., 2022; Zhu et al., 2025). This disrupts the root-soil interface, maximizes displacement, and results in peak energy dissipation. As the number of cycles increases, the root system adapts to external loads, with fewer adjustments and deformations. Soil particles rearrange more tightly, enhancing friction and cohesion (Stachew et al., 2021). As a result, the relative displacement at the root-soil interface decreases, and energy dissipation through friction diminishes, eventually reaching a stable state.

In summary, the results indicate that root systems experience significant energy dissipation under low-cycle loading conditions. However, provided that soil reinforcement performance is maintained, plant root systems can endure a certain degree of low-cycle loading without significant loss of anchoring capacity.

### 4.3 Bouc-Wen model analysis

This study enhances a hysteresis curve model based on the Bouc-Wen framework to fit the cyclic loading and unloading displacement-force curves at load amplitudes of 25%, 50%, and 75%. The improved model not only captures essential information about the load amplitude-displacement relationship during root cyclic loading but also incorporates additional shape parameters into the Bouc-Wen model to account for the elastic-plastic deformation characteristics of roots. This enhancement enables an accurate representation of the variation in root hysteresis curves under different load amplitudes. Furthermore, the fitting results of the improved model demonstrate that its accuracy remains consistent regardless of changes in root diameter. The model precisely fit root systems with diameters ranging from 1 to 10 cm, achieving an average R² value of 0.917. These findings highlight the Bouc-Wen model’s broad application potential in studying cyclic loads on plant root systems, though there is still significant room for improvement in terms of stability and accuracy. Future research could leverage machine learning and intelligent algorithms to estimate model parameters under cyclic loads, accelerating parameter identification and improving the fitting accuracy of results.

Additionally, the application of the improved Bouc-Wen model proposed in this study to root cyclic loads has considerable potential for further refinement. Given that the morphology and diameter of root systems, as well as indicators such as soil moisture content and soil type, have a significant impact on root anchoring capacity, these parameters could be incorporated as additional input variables during model training. This would increase the diversity of model features, enhance its reliability, and address potential overfitting issues. Therefore, determining model parameters is a critical step in optimizing prediction results, and this approach can be applied to studies on the nonlinear hysteretic behavior and the anchorage performance of plant root systems under cyclic loads. The enhancements to the Bouc-Wen model proposed in this study will serve as a valuable reference for understanding changes in the anchorage performance of root systems under cyclic loading conditions.

## 5 Conclusions

This study simulates the effects of natural cyclic loading on root systems in a controlled laboratory environment to investigate the response of *Betula platyphylla* root systems to cyclic loading in soil. A custom-developed root pull-out testing apparatus was employed to apply cyclic loading-unloading sequences to the root systems. Continuous force-displacement data were used to analyze the hysteresis behavior exhibited by root systems under different load amplitudes. Additionally, the study provides a comprehensive analysis of energy dissipation characteristics during cyclic loading and evaluates the applicability of an improved Bouc-Wen model for describing the hysteretic behavior of roots. The findings of this study have significant practical relevance, particularly for applications in slope stabilization and ecological engineering. By understanding how root systems respond to cyclic loading, this research can inform the design of more resilient vegetation for soil reinforcement in vulnerable landscapes. These results can be applied to enhance vegetation-based soil conservation strategies and improve the effectiveness of ecological engineering in regions prone to erosion and instability. Based on the experimental results, the following conclusions can be drawn:

1. Under different load amplitudes, there are two main failure modes. Under low-amplitude cyclic loads, the entire root system is completely uprooted after 100 cycles, while under high-amplitude cyclic loads, the root system is uprooted before completing 100 cycles.
2. The load amplitude is negatively correlated with the anchoring performance. Under cyclic load amplitudes of 25%, 50%, and 75%, the peak bearing capacity decreased by 12%, 17%, and 25%, respectively, after cyclic loading.
3. The energy dissipation of the root system increases with the load amplitude and decreases with the number of cycles. Under load amplitudes of 25%, 50%, and 75%, the energy dissipated during the 25th cycle is significantly lower compared to the first cycle, with reductions of 68.96%, 56.75%, and 53.21%, respectively.
4. The improved Bouc-Wen model effectively captured the hysteresis behavior of root systems under cyclic loading. Model fitting results demonstrated high accuracy, with an average coefficient of determination (R²) of 0.917 across all cases.

## Data Availability

The raw data supporting the conclusions of this article will be made available by the authors, without undue reservation.
